# Risk factors of hepatitis B transmission in northern Palestine: a case – control study

**DOI:** 10.1186/1756-0500-7-190

**Published:** 2014-03-28

**Authors:** Zaher Nazzal, Inam Sobuh

**Affiliations:** 1 Assistant Professor in Community Medicine, Faculty of Medicine and Health Sciences, An-Najah National University, Box 7,707, Nablus, Palestine; 2 MPH, Faculty of Graduate Studies, An-Najah National University, Nablus, Palestine

**Keywords:** Hepatitis B, Risk factors, Dental visits, Case–control study, Northern Palestine

## Abstract

**Background:**

The Hepatitis B (HB) infection is a significant health problem in Palestine, which is categorized as an HB virus moderate endemic area, with the HB carrier rate ranging from 2-6%. The aim of this study is to determine the risk factors of Hepatitis transmission in the northern areas of Palestine in order to help prevent and control this prevalent health problem.

**Methods:**

A case–control study was implemented to achieve the study objectives. One hundred HB virus seropositive cases and another 100 seronegative controls were included in the study. Univariate analysis and a logistic regression model were performed to examine probable risk factors of acquisition of HB infections.

**Results:**

Univariate analysis showed that HB case-patients were more likely to report having a history of blood transfusion, dental visits, hospitalization, Hejamat, sharing shaving equipments, intravenous drug use, or living abroad than controls were. The logistic regression model revealed a history of dental visits to be the most significant risk factor, (P value <0.001, OR 5.6; 95% CI 2.8-11.1).

**Conclusion:**

The presence of these risk factors emphasizes the need for both increasing the uptake of HB vaccine and implementing risk-targeted public health education. Development and enforcement of appropriate infection control guidelines for dental care services are important to prevent HB virus transmission as well.

## Background

The Hepatitis B (HB) virus infects people across the world; approximately one third of the world’s population has been exposed to the virus and an estimated 400 million people are chronically infected. Approximately 25% of those infected are at risk for mortality due to chronic liver disease or hepatocellular carcinoma (HCC) [1] Although HB infection is preventable through safe and effective vaccination and health education, it is associated with high morbidity and mortality and constitutes a high economic burden [2].

In the Middle East region, the prevalence of HB virus carriers among adults varies from less than 2%, as in Bahrain, to more than 15%, as in the Republic of Yemen [2]. Palestine has been categorized as an HB virus moderate endemic area where the HB carrier rate ranges from 2-6% [3]. These numbers were expected to decrease after the adoption of universal infant vaccination in 1992 into the Palestinian Expanded Program of Immunization, and the strong supervision policy that has been implemented by the Ministry of Health (MoH).

Identification of the demographic and behavioral determinants of the disease transmission is important to highlight the risk factors responsible for its spread and to increase awareness among people, specifically the other members of the households of those infected by HB. Additionally, this will help in identifying the target population in order to take appropriate measures to prevent and control the HB infection [4].

The aim of this study is to determine the risk factors of Hepatitis B (HB) transmission in the northern areas of the West Bank in an effort to obtain appropriate recommendations to limit its means of transmission and to decrease its morbidity and mortality.

## Methods

### Study design and population

A case–control study was conducted to evaluate the potential risk factors of HB transmission. All incident cases of HB infection during the period from December 2011 to December 2012 that met the inclusion criteria were enrolled in the study. The study was conducted at preventive medicine clinics in primary health care (PHC) departments in the northern areas of Palestine, where all newly discovered HB cases are notified.

The study population consisted of all newly-diagnosed (within the past six months from the time of the study) HB patients. Cases were diagnosed according to the applied Palestinian MoH policy in which confirmed diagnosis of acute HB is based on positive laboratory tests for anti- HBc IgM, or HBsAg and the patient is not known to have chronic HB [5]. The controls were members of the households of the cases who had no laboratory evidence of HB infection (seronegative), and were within 5 years of age of the cases.

### Data collection

An interviewer-administered questionnaire was used to achieve the study objective, which was constructed based on a number of different related studies [6-12]. It consisted of 36 questions, mainly concerned with the demographic characteristics of the participants, the source of HB detection and vaccination status, and HB transmission determinants.

All subjects were asked about personal behavior and health care services-related risk factors, such as intravenous drug use, sharing shaving instruments, sharing toothbrushes, shaving at a barber, Hejamat (phlebotomy, a procedure in traditional medicine of Palestine that is similar to bloodletting), getting piercings or tattoos, a history of Haj and Omra (Muslim pilgrimages), jail history (for more than three months), STD history, receiving blood, invasive surgery (including endoscopy procedure), hemodialysis, a history of dental visits, and a history of hospitalization.

Prior to data collection, the questionnaire was pre-tested with a convenience sample of 10 candidates of the study population to ensure the clarity, time, and ease of administration. Refinements were made on the basis of feedback from the pre-test. Those who participated in the pre-testing were excluded from the study sample.

Preventive medicine nurses interviewed both cases and controls. All nurses received standard training on how to interview the participants and how to clarify the questionnaire, emphasizing the importance of ensuring that each factor happened during the six months prior to detecting the HB infection.

### Statistical analysis

We used the Statistical Package for Social Sciences (SPSS) version 17 for data entry and statistical analysis. Descriptive statistics were computed to assess the personal characteristics of the participants. Univariate analysis was conducted using a Chi-square test with an odds ratio (OR) calculated for risk factors. Multivariate logistic regression was performed for variables found to be significant in univariate analysis. A *P-*value of <0.05 was considered to indicate statistical significance and a confidence interval was set at 95%.

The Institutional Review Broad of An-Najah National University (ANU) approved the study and approvals were additionally obtained from the Palestinian MoH. A signed consent form was obtained from all participants.

## Results

During the study period, a total of 100 patients were diagnosed with HB and accepted to participate in the study. The majority of the patients (72%) were in the age group 20–40 years old, male gender predominated among the cases (69%/31%) with male–female ratio is 3:1, and three-fourths of the patients were married. The majority of the cases were detected following blood donation, pre-employment examinations and pregnancy profile tests.

During the same period, 100 members of the households of the cases with negative tests for Hepatitis B who had come to the preventive medicine clinic agreed to participate in the study as part of the control group. Cases and the controls were matched by age (within 5 years of age). Table 1 shows social and demographic characteristics of participants. The two groups were comparable except for gender and occupation.

**Table 1:** Distribution of socio-demographic characteristics of the cases and controls

**Variable**	**Case (n = 100) frequency (%)**	**Control (n = 100) frequency (%)**	**P value**^**$**^
Age:			
▪ 20-30	34 (34)	41 (41)	0.394
▪ 31-40	38 (38)	41 (41)	
▪ 41-50	21 (21)	14 (14)	
▪ > 50	07 (07)	04 (04)	
Gender:			
▪ Male	69 (69)	45 (45)	0.001
▪ Female	31 (31)	55 (55)	
Residency:			
▪ City	22 (22)	21 (21)	0.984
▪ Village	58 (58)	59 (59)	
▪ Camp	20 (20)	20 (20)	
Level of education:			
▪ Illiterate	04 (04)	02 (02)	0.615
▪ Elementary & secondary	69 (69)	67 (67)	
▪ University	27 (27)	31 (31)	
Occupation:			
▪ Manual	14 (14)	09 (09)	0.005
▪ HCW	06 (06)	04 (04)	
▪ Military	14 (14)	02 (02)	
▪ Others*	66 (66)	85 (85)	
Marital status:			
▪ Married	75 (75)	71 (71)	0.524
▪ Unmarried	25 (25)	29 (29)	
Vaccination status:			
▪ Vaccinated	03 (03)	07 (07)	0.358
▪ Non-vaccinated	97 (97)	93 (93)	
Family size:			
▪ <5	66 (66)	71 (71)	0.447
▪ ≥5	34 (34)	29 (29)	
Monthly income (JD):			
▪ <500	68 (68)	69 (69)	0.87
▪ ≥500	32 (32)	31(31)	

*Others: students, employee, …etc. $Chi Squared test JD = Jordanian Dinar.

For the health-care services exposure risk factors, the univariate analysis revealed that a history of blood transfusion, dental visits, and hospitalizations were significantly associated with HB infection (P-value <0.05). On the other hand, it did not show significant differences between patients and controls regarding a history of dialysis, exposure to needle stick injury, and surgical operation (Table 2).

**Table 2:** Univarite analysis for the health-care services exposure risk factors of HB

**Variable**	**Case n (%)**	**Control n (%)**	**P value**	**OR**^**$ **^**(95% CI^)**
**History of dialysis:**				
▪ Yes	3 (3)	0 (0)	0.346^@^	2.0 (0.76-5.34)
▪ No	97 (97)	100 (100)		
**History of blood transfusion:**				
▪ Yes	24 (24)	9 (9)	0.004*	3.2 (1.4-7.28)
▪ No	76 (76)	91 (91)		
**History of needle stick:**				
▪ Yes	7 (7)	2 (2)	0.170^@^	3.7 (0.75-18.2)
▪ No	93 (93)	98 (98)		
**History of surgical operation:**				
▪ Yes	35 (35)	23 (23)	0.061*	1.8 (0.97-3.3)
▪ No	65 (65)	77 (77)		
**History of dental visits:**				
▪ Yes	75 (75)	29 (29)	<0.001*	7.4 (3.9-13.7)
▪ No	25 (25)	71 (71)		
**History of hospitalization:**				
▪ Yes	45 (45)	28 (28)	0.013*	2.1 (1.17-3.78)
▪ No	55 (55)	72 (72)		

*Chi Squared test^, @^Fisher exact test^, $^OR = Odds Ratio^, ^^CI: Confidence Interval.

The univariate analysis revealed a significant relationship between HB infection and some behavioral risk factors such as Hejamat, IV drug use, barber visits and living abroad for more than one year (P-value <0.05). On the other hand, it did not show a significant difference between cases and controls in regard to other personal behavioral risk factors such as getting tattoos or piercings, sharing pre-used tooth brushes, having a history of Haj and Omra, being in jail for more than three months, and having a history of STDs (Table 3).

**Table 3:** Univarite analysis of the personal behavior risk factors of HB

***Variable***	***Case n (%)***	***Control n (%)***	***P value***	***OR***^***$ ***^ ***(95% CI^)***
***History of Hejamat***				
• Yes	11 (11)	2 (2)	0.010*	6.1 (1.3-28.07)
• No	89 (89)	98 (98)		
***Tattoos***				
• Yes	5 (5)	1 (1)	0.097*	5.2 (0.59-45.4)
• No.	95 (95)	99 (99)		
***Piercing***				
• Yes	4 (4)	3 (3)	0.700*	1.36 (0.25-6.18)
• No	96 (96)	97 (97)		
***Sharing shaving equipment***^α^				
• Yes	29 (42.20	11(24.4)	0.049*	3.3(1.02-7.07)
• No	40 (58)	34 (75.5)		
***History of sharing toothbrush***				
• Yes	15 (15)	8 (8)	0.121*	2.0 (0.82-5.03)
• No	85 (85)	92 (92)		
***Intravenous drug use***				
• Yes	5 (5)	0 (0)	0.024^@^	2.05 (1.8-2.3)
• No	95 (95)	100 (100)		
***History of barber visit***^α^				
• Yes	29 (42.0)	10 (22.2)	0.032*	2.5 (1.1-5.9)
• No	40 (58.0)	35 (77.8)		
***History of Haj and/or Omra***				
• Yes	12 (12)	7 (7)	0.228*	1.8 (0.68-4.81)
• No	88 (88)	93 (93)		
***Living abroad (> one year)***				
• Yes	21 (21)	8 (8)	0.009*	3.0 (1.28-7.280)
• No	79 (79)	92 (92)		
***History of jail:***				
• Yes	9 (9)	3 (3)	0.074*	3.1 (0.84-12.2)
• No	91 (91)	97 (97)		
***History of STDs:***				
• Yes	1 (1)	0 (0)	0.316^@^	2.0 (1.75-2.31)
• No	99 (99)	100 (100)		

*Chi-square Test, ^@^Fisher exact test, ^$^OR = Odds Ratio, ^CI: Confidence Interval ^α^For male participant only.

The multivariate logistic regression model included all variables found to be significant in the univariate analysis; gender, occupation, blood transfusion, dental visits, hospitalization, Hejamat, sharing shaving equipment, intravenous drug use, barber visits and living abroad. Controlling for all the above-mentioned variables, the Logistic-Regression Model identified only dental visits to be significantly associated with HB infection (P-value < 0.001, OR = 5.6) (Table 4).

**Table 4:** Multivariate analysis for the Hepatitis B risk factors

**Variable**	**Case *n (%)***	**Control *n (%)***	***P *** **value**	***OR***^***$ ***^ ***(95% CI^)***
**Occupation:**				
* ▪ HCW*	4 (4)	6 (6)	0.66	1.4 (0.2-6.8)
* ▪ Soldier*	2 (2)	14 (14)	0.09	4.6 (0.8-28.2)
* ▪ Manual*	9 (9)	14 (14)	0.50	1.5 (0.5- 4.8)
* ▪ Others*^*1*^	85 (85)	66 (66)	0.38	1
**History of blood transfusion**	24 (24)	9 (9)	0.33	1.6 (0.6-4.6)
**History of dental visits**	75 (75)	29 (29)	<0.001	5.6 (2.8- 11.1)
**History of hospitalization**	45 (45)	28 (28)	0.43	1.4 (0.6 – 3.0)
**History of Hejamat**	11 (11)	2 (2)	0.20	3.5 (0.6 -20.2)
**Sharing shaving equipment**	29 (42.2)	11 (24.4)	0.48	1.4 (0.5 – 3.9)
**Intravenous drug use**	5 (5)	0 (0)	0.48	1.1 (0.2-4.2)
**History of barber visit**	29 (42.0)	10 (22.2)	0.33	1.6 (0,6 -4.37)
**Living abroad (> one year )**	21 (21)	8 (8)	0.19	2.0 (0.71- 6.1)

^$^OR = Odds Ratio.

^CI: Confidence Interval.

^1^Reference group.

## Discussion

The results showed that a number of behavioural and health care service-related factors put a person at risk for transmission of HB infection. All of these risk factors should be addressed by public health officials to formulate prevention and control measures. However, because the history of a dental visit was found to be the most significant risk factor associated with HB infection, there is an emergent need to intervene in this area.

The case group showed male predominance contrary to the control group. The literature shows that males are at a higher risk to contract the HB infection. Studies conducted in Egypt, Turkey and Brazil showed that the male gender was considered a risk factor for HB infection [10,11,13]. The majority of the study population was young adults, between the ages of 20 to 40 years. This result may be due to the rising incidence of risk factors for the HB infection toward the end of adolescence. This is also consistent with the allowed age range of blood donation, employment and the child bearing age of the females, the three most common sources of HB cases’ detection.

Our study revealed that only 5% of the study population reported being vaccinated against HB; 3% of the cases and 7% of the controls. This is consistent with what a study conducted in Egypt shows [11]. The low proportion of vaccinated participants (in both cases and controls) is an indication that more efforts should be dedicated to vaccinating adults born before 1992, especially those working in the fields that are related to the risk factors found in this study. This will not lessen the importance of other interventions, mainly those targeting the behavioral and the health care service-related risk factors, even among the vaccinated people. A considerable proportion of vaccinated people, for different reasons, fail to respond to the HB vaccine [14,15].

In terms of behavioral risk factors, the univariate analysis revealed higher proportion of cases with a history of Hejamat, sharing of shaving equipment, barber visits, intravenous drug use and living abroad. The fact that a higher proportion of cases had a history of Hejamat compared to controls is consistent with several studies done in developing countries [8,9,16]. This could be due to the fact that in Palestine non-professionals usually perform Hejamat, and there are neither rules nor guidelines to regulate and supervise this practice. Similarly, sharing of shaving equipment was found to be more prevalent among the case group.

A history of a barber visit (for shaving) was also found to be higher among cases, which is consistent with what has been found in an Iranian study [7]. Most barbers use the same blades and scissors for every customer and this practice increases the transmission of HB to both customers and barbers.

In terms of health care services-related risk factors, the univariate analysis shows that hospitalization, blood transfusions and dental visits are risk factors for HB infection. A recent history of hospitalization was also found to be a risk factor for HB transmission in Moldova [6], Brazil [13] and the KSA [17]. This result could indicate deficiencies in heath care workers’ (HCWs) knowledge and practice of universal precautions. Some studies on HCWs showed a lack of knowledge and practice of universal precautions, which led to exposing themselves and the patients nosocomial infections [18].

By using logistic regression analysis of the two groups, only the history of dental visit remained an independent risk factor for HB transmission. The majority of the HB cases gave a history of dental visits 75%, whereas only 29% of the control subjects had a similar history. This difference between the two groups was found to be highly statistically significant. Unhygienic dental care is a significant risk factor and plays a crucial role in HB transmission. Dental procedures have been shown to be associated with HB infection in many studies in countries such as in Jordan [12], Iran [7], Pakistan [9] and Moldova [6]. This has been attributed to the lack of sufficient knowledge and practice in clinical infection control.

A study conducted in Palestine in 2004 [19] showed that only 54.6% of dentists wear gloves when treating patients, 53.6% use the 70% alcohol solutions as a disinfectant agent and 83.2% of them use dry heat as a sterilization method, which has been proven to be inefficient. In Jordan, another study [20] conducted to assess the compliance of dentists in private dental clinics with infection control measures concluded that infection control practices are insufficient and reflect a great opportunity to transmit blood borne pathogens such as the HB virus through dental care. The HB virus can be transmitted in dental care clinics either through direct contact with blood and oral fluids, or through indirect contact with contaminated objects including instruments, equipment, and surfaces. These routes can transmit the disease from the dental staff to the patient, or vice versa, and from one patient to another [21].

In South Africa [22], a study conducted to assess the knowledge of infection control measures and compare it with actual practices in the field of dentistry found a significant difference between knowledge and practice. This finding shows the importance of supervision and enforcement of infection control measures in addition to raising awareness and increasing knowledge.

In this study, a number of measures were taken to mitigate possible limitations. To minimize recall bias, incident cases of HB were included in our study and all nurses who participated in data collection were trained on a standard method of data collection. The sample size was constrained by the number of people who detected HB during the study period. A multi-year study should be done to increase the sample size, allowing for more data to be collected about the rare risk factors of HB transmission found in other studies, such as STDs and IUD. Another possible limitation is that we were not able to assess the household effect as a risk factor since the controls were members of the same households as the cases. This is because the aim of this study was to explore more in depth the patients behavioral and health-care exposure risk factors for HB infection, as opposed to household effects as risk factors.

## Conclusion

Several personal behavioral and health care service-related risk factors were found to be more prevalent among the HB cases, such as blood transfusion, hospitalization, Hejamat, IV drug use, sharing shaving equipment, barber visits and living abroad. Specifically, a history of dental visits was found as the main independent risk factor for HB transmission, similar to other communities reported in other studies.

Further studies comparing knowledge and practice of infection control measures in the field of dentistry should be conducted in Palestine to understand the needed emphasis of a potential public health program to prevent the transmission of HB.

Instituting routine HB screening and vaccination for high-risk groups, adopting and enforcing infection prevention and control standards in Health Care Settings, especially dental clinics, and raising public awareness about the determinants of HB and its mode of transmission are necessary actions to prevent and control the disease.

## Competing interests

The authors declare that they have no competing interests.

## Authors’ contributions

*ZN* developed the study protocol, revised the methodology, contributed significantly to statistical analysis and interpretation of data, and developed the manuscript drafting. *IS* wrote the methods, lead the data collection, contributed significantly to statistical analysis and data interpretation and contributed to the manuscript drafting. Both authors read and approved the final manuscript
